# Guillain-Barré syndrome

**DOI:** 10.1093/emph/eoae020

**Published:** 2024-08-28

**Authors:** Nicholas Shedd, Peter Woods, Damon Hoad

**Affiliations:** Warwick Medical School, University of Warwick, Medical School Building, Coventry, CV4, UK; Warwick Medical School, University of Warwick, Medical School Building, Coventry, CV4, UK; Central England Rehabilitation Unit, Leamington Spa Hospital, Heathcote Lane, Heathcote, Warwick, Warwickshire CV34 6SR, UK; Warwick Medical School, University of Warwick, Medical School Building, Coventry, CV4, UK; Central England Rehabilitation Unit, Leamington Spa Hospital, Heathcote Lane, Heathcote, Warwick, Warwickshire CV34 6SR, UK

Guillain-Barré Syndrome (GBS) is the leading cause of acute flaccid paralysis globally. This immune-mediated condition of peripheral nerves often follows infection [1]. Symptoms are caused by antibody-mediated autoimmune demyelination and axonal destruction [2].

GBS is clinically heterogeneous with multiple variants. Acute inflammatory demyelinating polyradiculoneuropathy is the classical presentation and the most common form of GBS in North America and Europe. It consists of progressive ascending limb weakness, associated with depressed or absent reflexes [1].

Variants preferentially targeting the axons of nerves include acute motor axonal neuropathy, and acute motor and sensory axonal neuropathy. Other rare variants include Miller Fisher Syndrome, paraparetic GBS, pharyngeal-cervical-brachial weakness, bilateral facial palsy with paresthesia, and Bickerstaff brainstem encephalitis [1].

Worldwide, GBS accounts for around 100 000 new cases annually. It is more common in men and increases in prevalence with age [1]. Treatment primarily involves supportive care, with early administration of immunoglobulins or plasmapheresis in some. In severe cases, ventilation may be required [1, 2].

## Evolutionary perspectives

GBS is associated with multiple pathogens including Campylobacter jejuni, zika, cytomeglovirus, and severe acute respiratory syndrome coronavirus 2 (SARS-CoV2) [1]. In GBS, infection is believed to induce autoimmune processes due to cross-reactivity of neuronal and pathogenic proteins [2]. This phenomenon is known as molecular mimicry and GBS is one of the earliest and best-supported examples [2].

Molecular mimicry emerges from selection pressure on pathogens to avoid the host immune system. This pressure has led to the convergent evolution of pathogen and host epitopes [3]. Understanding the evolutionary pressures leading to the autoimmune processes in GBS explains the variety of triggering pathogens and clarifies the heterogeneous presentations of this syndrome.

Variation in GBS subtype is influenced by the antecedent pathogen. Zika is associated with sensorimotor demyelinating GBS with frequent facial palsy. Zika outbreaks in the early and mid-2010s resulted in an increase in GBS incidence and a change in global subtype prevalence [4]. These outbreaks demonstrate that emerging pandemics and new zoonoses can influence the presentation of GBS, likely due to differing neuronal targets across pathogens. This variety in molecular targets inducing cross-reactivity may influence the presentation and severity of GBS; and impact the need for escalation of treatment.

Recently, vaccination against SARS-CoV-2 has been linked to the development of GBS, particularly in regard to adenovirus-vector vaccines [5]. This heightened risk suggests potential cross-reactivity with vaccination vector epitopes, highlighting the importance of considering GBS in vaccine design. Infection with SARS-CoV-2 has also been linked to GBS, via proposed methods such as those detailed by Stoian *et al*. [6]. This may present a conflict in vaccine design: balancing the need to limit GBS-inducing viral strains with iatrogenic GBS cases.

In addition to immune triggers, several genes related to dysregulation and dysfunction of immune cells have been linked with an increased lifetime likelihood of developing GBS [7]. These genes likely predispose to immune cross-reactivity.

## Future implications

Viewing GBS through an evolutionary lens could unlock innovative treatments, public health strategies, and prognostic insights while mitigating vaccine-related GBS cases.

Investigation into therapies targeting antiganglioside antibodies associated with GBS, such as eculizumab, are underway, and have yielded mixed results. Phase 2 trials of eculizumab did not meet the predefined response rate. However, a higher proportion of the eculizumab-treated group were able to walk independently after 4 weeks (61% vs 45%) [8].

**Figure 1.**
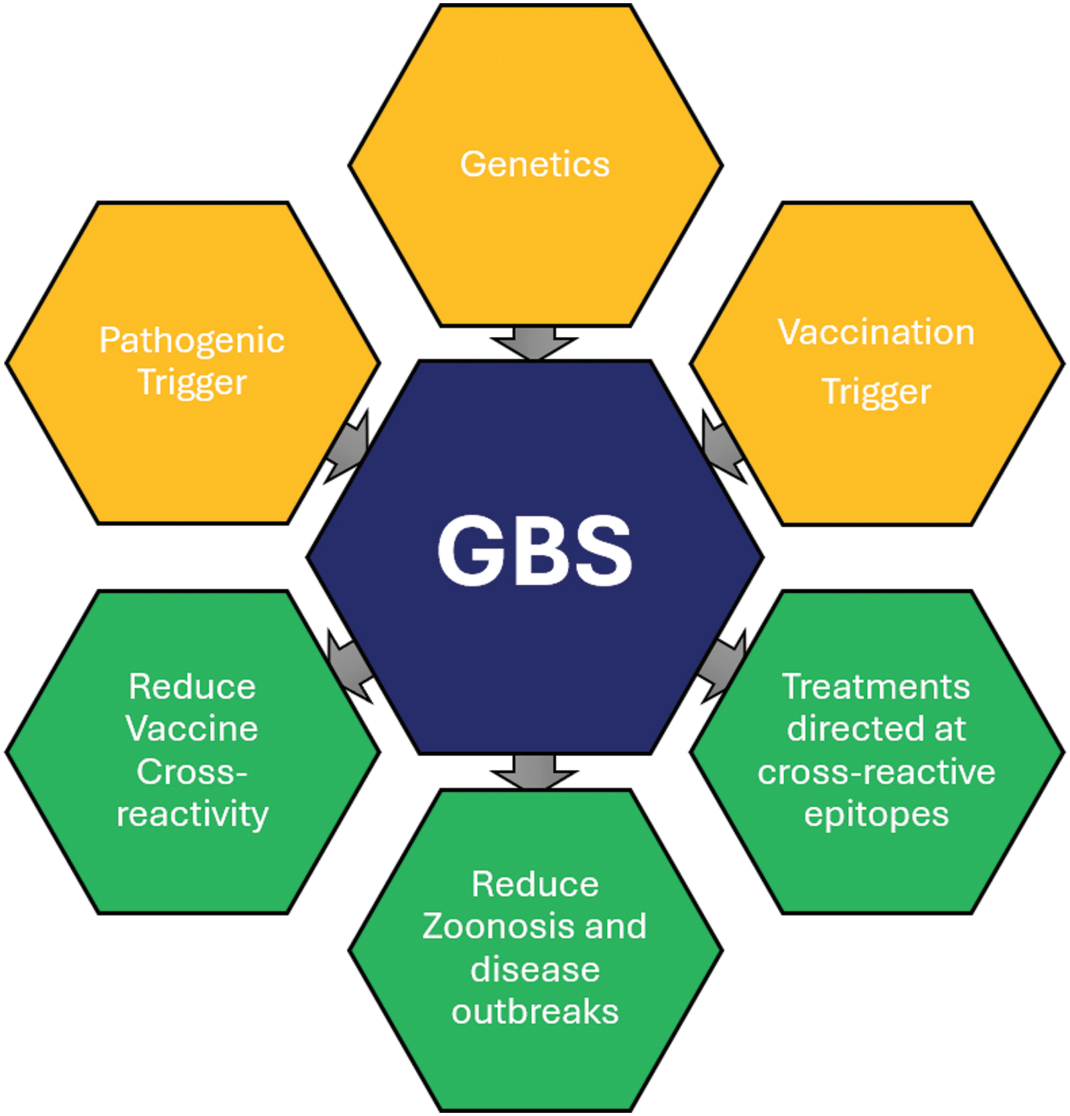
Factors that can increase (Yellow) or decrease (Green) the prevalence of GBS

Vaccination programs targeting GBS-associated pathogens could reduce its incidence. These may have to weigh the risk of vaccine-induced GBS versus potentially detrimental selection pressures when preferentially targeting pathogen epitopes which do not exhibit molecular mimicry. However, careful vaccine design may help to limit GBS cases (Fig. 1).

Awareness that pathogen co-evolution leads to GBS is especially relevant in regard to novel or zoonotic pathogens, as has been made clear by SARS-CoV-2. Recognition and reduction of infections with the potential to induce antibody cross-reactions could lead to a decrease in the burden caused by GBS globally.
